# The Role of Electrophysiological Workup in Lumbar Spinal Canal Stenosis (LSCS): A Systematic Review

**DOI:** 10.1097/BRS.0000000000005585

**Published:** 2025-12-11

**Authors:** Guichande Duarte, Markus Hupp, José Spirig, Mazda Farshad, Martin Schubert, Armin Curt, Patrick Freund, Carl M. Zipser

**Affiliations:** a Department of Neurology and Neurophysiology, Balgrist University Hospital, University of Zurich, Zurich, Switzerland; b University Spine Center, Balgrist University Hospital, University of Zurich, Zurich, Switzerland; c Department of Spine Surgery, Balgrist University Hospital, University of Zurich, Zurich, Switzerland

**Keywords:** neurophysiology, lumbar spinal canal stenosis, electromyography, gait assessments

## Abstract

**Study Design.:**

Systematic literature review.

**Objective.:**

Update on diagnostic utility of electrophysiology in lumbar spinal canal stenosis (LSCS).

**Summary of Background.:**

LSCS is a highly prevalent degenerative spine condition characterized by neurogenic claudication, radicular pain, and muscle weakness. While lumbar spine MRI is the imaging modality for detecting spinal canal narrowing, it correlates poorly with clinical symptoms. Electrophysiological methods, including electromyography (EMG), nerve conduction studies (NCS), and evoked potentials (MEP and SEP), may provide complementary information on neural dysfunction. Current guidelines support paraspinal electromyography (EMG) mapping for symptomatic patients with imaging confirmed stenosis (grade B). In contrast, the diagnostic value of other electrophysiologic tests in LSCS remains uncertain.

**Methods.:**

A systematic literature search was conducted in Medline and Embase for original studies on LSCS between 2020 and 2024. Two independent reviewers screened studies for inclusion. The extracted data was synthesized qualitatively. Study quality was assessed using the Robins-V2 tool. PROSPERO registration (CRD42024622427).

**Results.:**

Thirteen studies met the inclusion criteria; study quality was moderate. Needle EMG of the limbs was evaluated in 23% of studies to detect denervation as a sign of radiculopathy. Twenty-three percent of the studies examined tibial nerve SEP or cauda equina MEP conduction time for lesion localization, with varying findings and utility for diagnosing LSCS. Surface EMG was investigated in 31% of studies and revealed significantly altered muscle activation patterns and compensatory gait adaptations in LSCS.

**Conclusion.:**

There is an increasing number of studies combining surface EMG with gait assessments and tasks. This approach is interesting for being noninvasive, with clinical utility to be further determined. On the basis of previous guidelines, paraspinal mapping is considered the gold-standard electrophysiological diagnostic tool. Interestingly, there were no recent studies on paraspinal mapping, indicating a shift to alternative methods.

Lumbar spinal canal stenosis (LSCS) is a highly prevalent degenerative spinal condition characterized by the narrowing of the spinal canal,^1^ often accompanied by constriction of the lateral recesses or neural foramina in the lumbar region.^2^ This anatomic narrowing can compress neural structures, resulting in lower back pain, neurogenic intermittent claudication (NIC), muscle weakness, gait disturbances, and, in severe cases, bladder and bowel dysfunction.^2–5^ LSCS is a leading indication for spinal surgery in older adults, with incidence increasing after the age of 65.^2,6–8^ In the United States of America alone, more than 200,000 cases are reported annually.^9,10^

Diagnosing LSCS is challenging and relies on a combination of clinical^8,11,12^ and radiologic findings.^13,14^ Magnetic resonance imaging (MRI) is the gold standard for detecting anatomic narrowing^15^; however, studies show that up to 30% of older adults may exhibit imaging evidence of spinal stenosis despite being asymptomatic.^4,16–21^ Moreover, the correlation between radiographic severity and clinical presentation is usually poor, particularly in multilevel stenosis. The clinical-imaging correlation can be further complicated by anatomic variants of lumbosacral nerve roots found in 5% of patients.^22^ This gap has led to a growing interest in using neurophysiological techniques as complementary tools for assessing clinically relevant neural compromise. These techniques include electromyography (EMG), nerve conduction studies (NCS), and evoked potentials.^23^ They help quantify and localize nerve damage, and distinguish symptomatic from incidental findings, especially in cases where imaging is inconclusive.

Current guidelines from the German Society for Neurology^24^ and the North American Spine Society (NASS)^25^ recognize a limited but supportive role for electrophysiological diagnostic in LSCS. Paraspinal mapping has demonstrated diagnostic utility in confirming the diagnosis, quantifying the severity of nerve damage, and localizing affected spinal levels.^26,27^ Nevertheless, the diagnostic value of specific modalities such as F-waves, H-reflexes, somatosensory (SEPs), and motor evoked potentials (MEPs) remains inconclusive.^28,29^

This systematic review provides an overview of the diagnostic utility of electrophysiological techniques for LSCS, focusing on evidence from the past five years. The aim is to highlight emerging techniques, identify gaps in current evidence, and guide future research to improve the diagnosis and management of LSCS.

## MATERIALS and METHODS

### Search Methods and Strategy

This systematic review was conducted in accordance with the PRISMA (Preferred Reporting Items for Systematic Reviews and Meta-Analyses) guidelines.^30^ Registration on PROSPERO (CRD42024622427). A comprehensive literature search was performed in Medline (PubMed) and Embase, restricted to the last five years (2020–2024) to capture recent advances in electrophysiological assessment of LSCS. Earlier studies (*i.e.*, 2008–2018) were included by the 2011 NASS Guidelines^25^ and by Zileli *et al*.^31^ A supplemental search for 2019 revealed studies that mostly overlap with our findings from 2020 to 2024. The research question was structured using the PICO framework (Table 1). Two reviewers independently screened all abstracts, and full texts were retrieved for eligibility assessment. If there were unresolved discrepancies between the two reviewers a third reviewer independently assessed the study to reach a final decision. Reference lists of eligible studies were manually screened to identify additional relevant publications not captured in the initial search. The search strategy applied was:

1. ((Lumbal stenosis[Title/Abstract]) OR (Lumbar Stenosis[Title/Abstract]) OR (Lumbar Spinal Stenosis[Title/Abstract]) OR (Lumbal spinal Stenosis[Title/Abstract]) OR (Lumbar Spinal Canal Stenosis[Title/Abstract]) OR (Lumbal Spinal Canal Stenosis[Title/Abstract])).
2. (Electrophysiology[Title/Abstract]) OR (EMG[Title/Abstract]) OR (Electromyography[Title/Abstract]) OR (Neurophysiology[Title/Abstract]) OR (nerve conduction studies[Title/Abstract]) OR (evoked potentials[Title/Abstract])).
3. diagn* #1 AND #2.

**TABLE 1:** The PICO Model Was Used to Construct the Research Question

Population (P): Patients with lumbar spinal canal stenosis (LSCS), diagnosed or suspected, adults (18+ yr)
Intervention (I): Electrophysiological techniques such as electromyography (EMG), motor evoked potentials (MEP), somatosensory evoked potentials (SSEP), and nerve conduction studies (NCS)
Comparison (C): Comparison of the effectiveness of different electrophysiological techniques against each other or against the gold standard (lumbar spine MRI)
Outcome (O):
Diagnostic accuracy (sensitivity and specificity)
Clinical relevance and prognostic value
Limitations and challenges associated with each technique
Identification of gaps in the current literature
Key Question (KQ) 1: Which neurophysiological examinations have been investigated in LSCS and how was their interrelation explored?
KQ 2: What is the current evidence on the role of NCS, EMG, MEP, SEP, F-waves, H-reflex, and paraspinal mapping in diagnosing LSCS?
KQ 3: How do these electrophysiological methods contribute to the clinical management of LSCS?
KQ 4: What limitations and challenges are associated with the use of these techniques in LSCS?
KQ 5: Are there gaps in the literature that require further investigation regarding the standardization and sensitivity of these methods?

Inclusion criteria: adults aged 18 years or older; confirmed diagnosis of LSCS; use of electrophysiological testing and neuroimaging; original research articles published in English. Exclusion criteria: studies where LSCS was not the primary condition; studies with unclear methodology or lacking clinical relevance; intraoperative electrophysiological studies; reviews; uncontrolled case series or case reports.

### Quality Assessment

Risk of bias was assessed using the ROBINS-I-V2 tool (Risk-Of-Bias-In-Non-randomized-Studies - of Interventions).^32^ Two reviewers independently screened the included articles. Discrepancies were resolved by consensus or third-party arbitration.

### Data Extraction

Key parameters from the included studies were extracted. To gain insight into diagnostic accuracy particular attention was given to the characteristics of the study population, electrophysiological modalities used, and LSCS severity as determined by imaging. Where possible, diagnostic metrics were statistically pooled; otherwise, findings were synthesized qualitatively.

## RESULTS

### Study Inclusion

**Figure 1:**
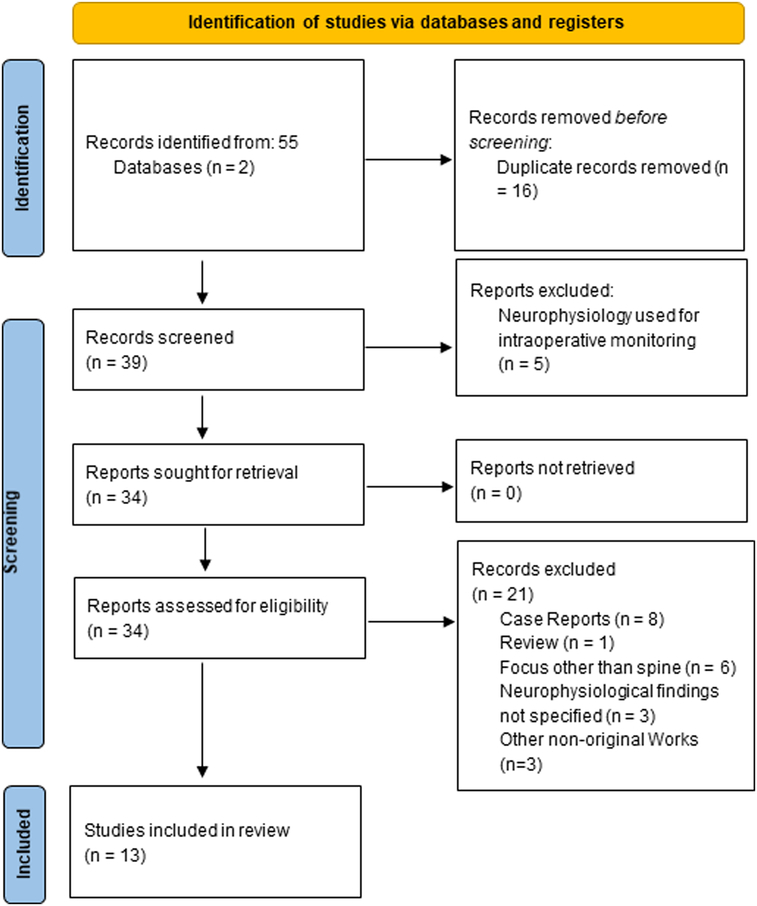
PRISMA flowchart of study inclusion.

Overall, 55 references were identified. After screening, 34 articles were retrieved for full-text review (Figure 1), and 13 met the inclusion criteria and were included in the final analysis (Table 2). Due to the heterogeneity of neurophysiological tests, study population, and reference standards, statistical pooling was not possible. Therefore, results were summarized in a qualitative manner.

**TABLE 2:** Study Overview

First Author, Year, Study Design	Characteristics of Participants: Symptoms/ Time of Symptoms/ Scheduled to Surgery?	Radiologic Classification of Stenosis and Diagnostic Criteria	Sample Size	Mean Age LSCS Years (Range); Sex (Males/ Females)	Electrodiagnostic Tests Investigated	Comparators	Key Findings
Ekström *et al*, 2020, prospective study^42^	Degenerative sLSCS confirmed on MRI/ NIC >6 mo/scheduled for decompression surgery	MRI-confirmed degenerative LSCS	12	67 (±5.3); 4M/8F	sEMG of erector spinae bilaterally at level L2 and L4; muscle tissue oxygen saturation (MrSO2) with near-infrared spectroscopy (NIRS) at L4	Preoperative *vs.* postoperative measurements NIRS, as well as the intensity of the leg and back pain and perceived exertion	Regional MrSO2 decreased during loading, returned to baseline during recovery. Both low back and leg pain were reduced after surgery
Park *et al*, 2020, retrospective study^36^	Upper LSCS with CSA <100 mm² on MRI (four patterns of neurogenic findings identified: mPPN, bipoly, unipoly, monorad)/not specified/not specified	MRI CSA of dural sac <100 mm²	14	67.1 (±7.7); 11M/3F	NCS, F-responses, H-reflexes, Needle EMG (lower extremity muscles, lumbar paraspinal muscles, and proximal muscles)	Structural MRI findings *vs.* electrophysiological findings in between neurological and structural levels	L5, S1 radiculopathies caused by upper LSCS (L1/2 to L3/4) show a discrepancy between structural and neurological levels. Bilateral radiculopathy is more common than unilateral. CMAP abnormalities were frequently observed
Yang *et al*, 2020, retrospective^38^	Patients with unilateral buttock/leg symptoms with claudication and MRI-confirmed LSCS at L4–5	MRI antero-posterior diameter (<10 mm for absolute, <13 mm for relative stenosis), CSA, LID, CSA-NF, SZW	40	62.1 (±14.8); not reported	L5 Dermatomal SEP (P40 latency in L5 dermatomes)	Comparison of normal *vs.* delayed P40 latency with radiologic severity of stenosis (APD, LID, *etc.*)	L5 Dermatomal SEP latency negatively correlated with LSCS severity. L5 Dermatomal SEP latency negatively correlated with APD (*r*=−0.539) and LID (*r*=−0.459). In relative stenosis, latency positively correlated with CSA-NF (*r*=0.371). LID significantly affected latency (β=−0.930)
Lin *et al*, 2020, prospective observational cohort study^41^	At least mild LSCS on MRI, CT, NIC, no lumbar spine surgery or ESI in the past 6 mo / not specified /not specified	MRI central canal stenosis	11	64.1 (±10.8); 11M	EMG with needle EMG and Biomarkers analyzed: MCP-1, RANTES, IL-1b, etc.; Inflammatory cytokines in lavage and serum studied	Predicting pain and functional response to epidural steroid Injections for LSCS with biomarkers and electromyography	High MCP-1 serum levels positively correlated with 2 mo patient satisfaction. Radiculopathy signs on needle EMG correlated with PDQ Score improvement at 1 mo
Kim, 2020, Prospective case-control study^31^	LSCS defined by MRI criteria, symptomatic with grade >1 and moderate stenosis at L2-5 segments / not specified / no surgery planned	Moderate stenosis at L2-5 segments on MRI	17	LSCS 66.1 (±8.0), Control 50.5 (±6.0); 3M/14F	sEMG - Gluteus medius, tensor fasciae latae, quadriceps femoris	Comparison of different gait patterns (normal, adducted and abducted gait) (femorotibial angle and sEMG) in LSCS patients vs healthy controls	LSCS patients show higher hip abductor activation in all gait patterns. Patients had wider stride width and femorotibial angles closer to varus. Increased pain scores in the LSCS group, especially during the abducted gait
Nagao *et al*, 2020, observational, cohort study^39^	LSCS with NIC / several months of conservative treatment without success before surgery / yes	MRI or CT myelography/obvious compression on the dural sac confirmed through MRI	149	Cauda equina-type 70,3 (±8.7), radicular-type 70,3 (±9.0); mixed-type 69,5 (±8.3)	CECT, compound muscle action potentials, F-waves, MEPs from Abductor hallucis, Motor nerve conduction and F-waves	Comparison of CECT across cauda equina-type, radicular-type, and mixed-type NIC	CECT significantly prolonged in cauda equina-type and mixed-type LSCS; negatively correlated with dural sac CSA
Shin and Yoo, 2021, observational study^32^	Elderly female patients with LSCS / not specified / not specified	Diagnosed with LSCS by a surgeon based on CT or MRI	9	77.0 (±4.7); 9F	sEMG of gluteus medius, vastus lateralis (stance limb)	Differences in sagittal kinematics and muscle coordination during SU and SD in patients with LSCS	SU tasks require higher trunk angles and isolated gluteus medius activation. SD tasks are more difficult due to reduced leg extensor output
Cai *et al*., 2021, exploratory study^40^	Group A: rLSCS + sLSCS with NIC; B: rLSCS without NIC; C: Control group without LSCS findings or symptoms / not specified / not specified	LSCS: central canal AP diameter =12 mm or CSA <80 mm²	17 (Group A-9, B-5, C-3)	A 68.6 (±5.9), B 65.6 (±9.1), C 69.0 (±1.7); 17M	MUNIX, NCS, surface and needle EMG according with affected myotome	Correlation with Pain and functional scores	No differences in MUNIX values across the three groups (individual muscles or combined). MUNIX values did not correlate with pain or functional measures
Nüesch *et al*., 2022, single-center cross-sectional observational study	Patients with clinical and MRI diagnosis of sLSCS, with BMI <35 kg/m² and no prior decompression surgery / >6 mo/ yes	MRI Schizas classification	20	70.4 (±8.5); 8M/12F	sEMG, inertial sensors (gluteus medius, erector spinae, multifidus)	Do muscle activation patterns together with sagittal joint kinematics differ between sLSCS and healthy controls and do these differences correlate with clinical scores?	Patients walked 0.26 m/s slower than controls. Higher midstance activation of multifidus, erector spinae, and gluteus medius in patients *vs.* controls. Clinical scores did not correlate with mGPS or EMG-profile scores within patients
Matsukura *et al*., 2023, retrospective multicenter study^37^	MRI-confirmed LSCS affecting cauda equina or conus/epiconus region L5/S1 Radikulopathie/not specified/not specified	Moderate to severe stenosis; classified into central or lateral types based on Arnoldi *et al*.	18	66.8 (±6.6); 15M/3F	Tibialis SEPs P15 and N21 potentials evaluated for localization of lesions, F-wave, Needle EMG, NCS	Comparison of localizing and nonlocalizing SEP findings and F-wave abnormalities with LSCS + needle EMG for segmental involvement	Segmental SEP sensitivity (67%) is higher than F-wave (36%) for localization; not statistically significant. Delayed P38 latency had 28% sensitivity. Segmental SEP better localizes lesions at the cauda equina or conus/epiconus levels compared with the F-wave
Rustom *et al*., 2024, retrospective cohort study^35^	Patients with low back pain and suspected LSCS or LNS/not specified/not specified	MRI; radiographic LSS/LNS evaluated for severity (none = 0, mild = 1, mild/moderate = 2, moderate = 3, moderate/severe = 4, severe = 5).	109	Not reported	Needle EMG Levels Th11-S1	Correlation between LSCS/LNS severity on MRI and needle EMG evidence of radiculopathy	No significant association between LSCS/LNS MRI severity and EMG findings; MRI not a reliable predictor of Needle EMG-confirmed radiculopathy
Park *et al*., 2024, retrospective cohort^33^	rLSCS on MRI and underwent EDX with 1 yr previously/not specified/not specified	≥1 intervertebral disc level with central canal stenosis (CSA <100 mm^2^ on MRI). rLSCVS severity by Lee *et al*.	193	No RNR, N: 78, age 65,83 (±10.72); With RNR, N: 115, Age 71,94 (±8.41); 76M/117F	Needle EMG - muscle selection according to Myotome within clinical presentation	Presence of RNRs and denervation potentials (ASA on EMG)	Severe RNRs significantly increase the probability of ASA on EMG. ASA probability increases with: advanced age (*P* < .001), longer symptom duration (*P* = .009), smaller dural sac CSA at the stenotic level (*P* < .001), and higher ASA frequency (*P* < .001)
Urbanschitz *et al*., 2024, observational study^30^	sLSCS with NIC/ >6 mo/yes *same study sample as Nüesch *et al*., 2022	MRI Schizas classification	20	70.4 (±8.5); 8M/12F	sEMG, inertial sensors / Gluteus medius, erector spinae, multifidus	Walking stress in LSCS *vs.* asymptomatic controls	No amplification of differences postwalking stress; gait/muscle activity differences exist between sLSCS and controls but do not increase after walking

* Same sample, different publications from Nüesch *et al*., 2022 and Urbanschitz *et al*., 2024^30^.

ASA indicates abnormal spontaneous activity; APD, antero-posterior diameter; CECT, Cauda Equina conduction time; CSA, cross-sectional area; CSA-NF, cross-sectional area nerve root foramina; EMG, electromyography; LID, ligamentous interfacet distance; LSCS, lumbar spinal canal stenosis; LNS, lumbar neuroforaminal stenosis: MUNIX, motor unit number index; MRI, magnetic resonance imaging; NCS, nerve conduction studies; NIC, neurogenic intermittent claudication; PDQ, Pain Disability Questionnaire; rLSCS, radiologic signs suggestive of LSCS; RNR, redundant nerve roots; sEMG, surface electromyography; SEP, somatosensory evoked potentials; sLSCS, symptomatic LSCS; SU, step up task; SD, Step down task.

### Analysis of Bias

**Figure 2:**
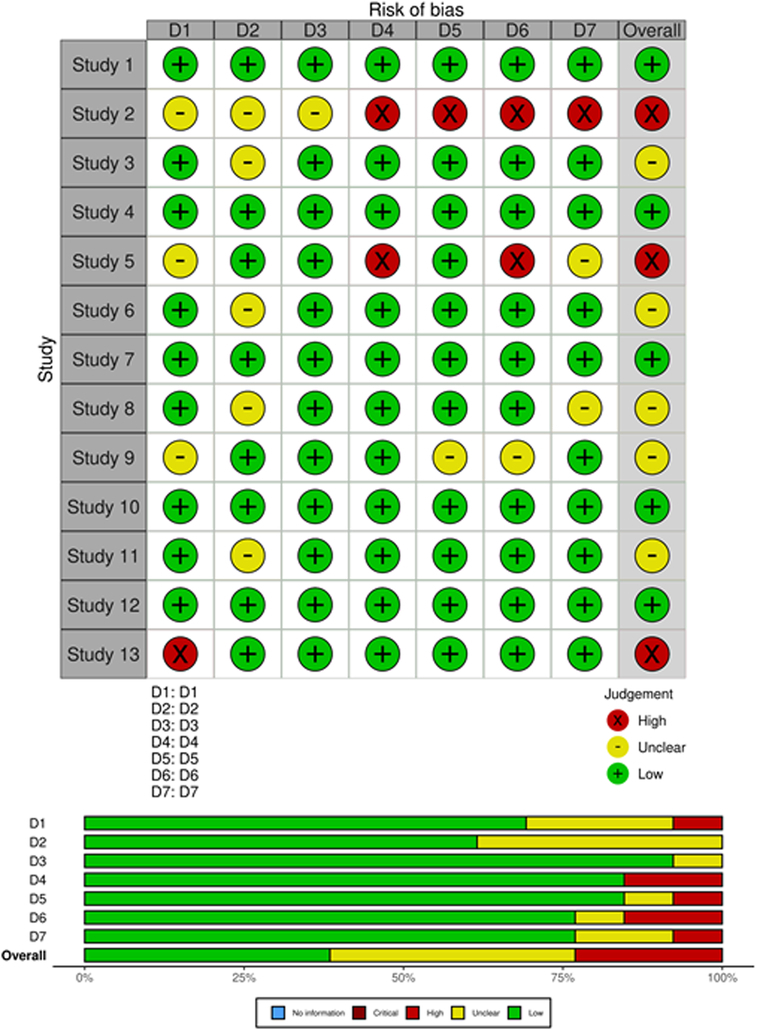
“Traffic Light” plots of the domain level judgments for each individual result and weighted bar plots of the distribution of risk-of-bias judgements within each bias domain.

Overall, study quality ranged from satisfactory to moderate (Figure 2); five studies were rated as having low risk of bias. Common concerns included selection and diagnostic bias.

### Qualitative Synthesis of the Literature

**Figure 3:**
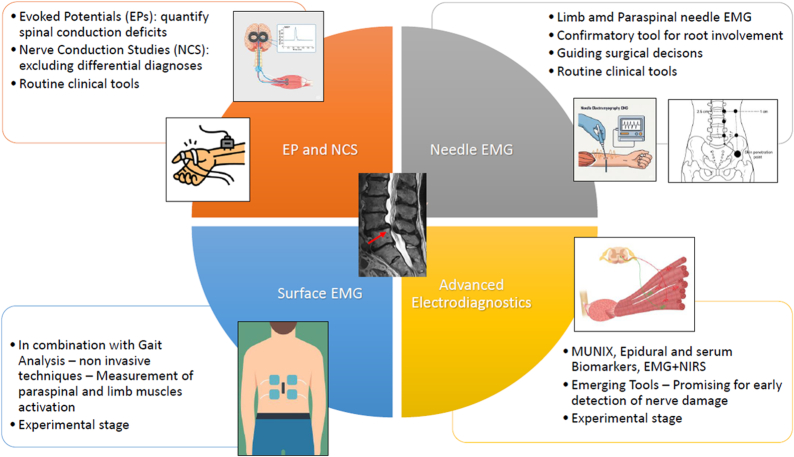
Overview of included methodologies (pictograms). The central image depicts a representative case of lumbar spinal canal stenosis (LSCS), illustrated by a sagittal T2-weighted MRI scan. The other images were generated using artificial intelligence (ChatGPT version 4.0).

Four distinct clusters were identified: Kinematic gait analysis combined with surface EMG (sEMG); conventional needle EMG studies; NCS, MEPs, and SEPs; as well as exploratory studies with advanced electrodiagnostic tools (Figure 3). Most studies combined surface EMG and kinematic gait analysis in LSCS patients (31%,4/13).

### Kinematic Gait Analysis Combined With sEMG

In a prospective study by Nüesch *et al*,^33^ 20 LSCS patients (predominantly Schizas grade C) were compared with 19 healthy controls. Higher sEMG activity was reported in the multifidus, erector spinae, and gluteus medius (Gmed) during midstance in LSCS patients. Gait in LSCS patients was characterized by reduced speed, cadence, and stride length, with a prolonged stance phase. However, these parameters did not correlate with pain or disability scores. A follow-up study by Urbanschitz *et al* ^34^ using a 30-minute walking stress test on the same patients revealed no functionally relevant changes in gait parameters. Still, LSCS patients with increased pain demonstrated higher sEMG activation, while linearity was not further analyzed.

In a prospective case-control study investigating 17 LSCS patients and 20 healthy controls, Kim *et al* ^35^ reported increased activation of the quadriceps and hip abductors in LSCS patients across various gait tasks.

Shin *et al* ^36^ assessed nine female LSCS patients performing a step‑up and step‑down task—height-of-step=19.5 cm—recording trunk/limb kinematics, as well as Gmed and vastus lateralis sEMG. The step‑down task showed greater Gmed and Vastus Lateralis activation, reduced hip and knee range of motion, and altered trunk and pelvic kinematics, compared with the step‑up task.

### Needle- EMG Studies

The correlation between needle EMG findings in the lower extremities and the level and severity of stenosis on MRI was investigated in 3/13 studies (23%). In a retrospective study of 193 patients, Park *et al* ^37^ found that detection of abnormal spontaneous activity (ASA) on needle EMG correlated with the presence and severity of redundant nerve roots (RNRs) on MRI scans. RNRs are nerve roots that become elongated, tortuous, and thick due to lumbar constriction.^38^

In a retrospective cohort study of 109 LSCS patients, Rustom *et al* ^39^ found no significant association between denervation on EMG and radiographic evidence of central canal or foraminal stenosis on MRI.

Park *et al*,^40^ in a retrospective review of 14 patients with upper lumbar stenosis (L1/2, L2/3, L3/4), found discrepancies between the radiologic levels of stenosis and neurophysiological findings. Bilateral EMG abnormalities were predominantly localized to L5 and S1 myotomes, despite structural compression occurring at higher lumbar levels. Reduced compound muscle action potentials were present in 10 patients, delayed F-wave latencies in five, and absent H-reflexes bilaterally in seven and unilaterally in three. RNRs on MRI were present in 13/14 patients and associated with chronic reinnervation on needle EMG.

### NCS, MEPs, and SEP

In 23% of reviewed studies (3/13) the role of evoked potentials was assessed. Matsukura *et al*,^41^ retrospectively evaluated the diagnostic utility of segmental tibial nerve SEPs with P15, N21, and P38 components in 18 patients with MRI-confirmed LSCS involving the cauda equina or conus/epiconus. The study demonstrated that segmental SEPs could localize abnormalities in 67% of cases, significantly outperforming delayed P38 latency (28%), and showing a trend towards higher sensitivity compared with N21 abnormalities (39%) and F-wave findings (36%). Segmental SEPs revealed localizing abnormalities in symptomatic LSCS patients without clinical sensory symptoms.

In a retrospective study, Yang *et al* ^42^ analyzed L5 dermatomal SEPs in 40 patients with MRI-confirmed LSCS at the L4–L5 level. While SEP latency did not differ based on radiologic severity, it was inversely correlated with anteroposterior diameter of the central canal (*r*=–0.539) and ligamentous interfacet distance (*r*=–0.459).

Nagao *et al* ^43^ investigated the relationship between cauda equina conduction time (CECT) and type of NIC in 149 patients scheduled for LSCS surgery, categorized into cauda equina-type (n=67), radicular-type (n=29), or mixed-type (n=53). Mean CECT was prolonged in cauda equina (5.6±1.1 ms) and mixed types (5.1±0.9 ms), compared with radicular type (4.0±0.9 ms).

### Advanced Electromyography Tools

Three studies (23%) explored advanced electromyographic techniques in LSCS.

Motor unit number index (MUNIX) is an electrophysiological method used to estimate the number of viable motor neurons that innervate a muscle. Cai *et al* ^44^ investigated MUNIX in 17 male participants across three groups: symptomatic LSCS, asymptomatic radiologic LSCS, and healthy controls. Total MUNIX scores across tibialis anterior, extensor digitorum brevis, and abductor hallucis did not differ between groups or correlate with functional disability or pain severity.

In a prospective observational cohort study, Lin *et al* ^45^ investigated the predictive value of serum and epidural lavage biomarkers, as well as Needle EMG, in determining clinical outcomes following interlaminar epidural steroid injection (ESI) in 11 patients with clinical and MRI-confirmed LSCS. Higher serum MCP-1 were strongly correlated with patient satisfaction at 2 months post-ESI (*r*=−0.915), while EMG-confirmed radiculopathy was inversely associated with short-term functional improvement (Pain Disability Questionnaire score; *r*=–0.828).

Ekström *et al*.^46^ investigated muscle function and oxygenation in 12 LSCS patients before and after laminectomy using a combined lumbar sEMG and near-infrared spectroscopy (NIRS) protocol during an isometric lumbar extension. sEMG amplitude was lower at the L4 level than at the L2 level, with no differences between preoperative and postoperative measurements. Muscle tissue oxygen saturation decreased during isometric loading, reflecting muscular exertion, and recovery to baseline after surgery.

## DISCUSSION

### Summary of Main Findings

Studies on electrophysiological diagnostics in LSCS conducted in the past five years demonstrate a wide range of established methodologies and novel diagnostic strategies providing insights that enhance diagnostic accuracy and sensitivity in LSCS. Key areas of interest include:

1. The role of needle EMG in confirming and localizing radiculopathy in LSCS.
2. The correlation between symptoms, MRI stenosis, and electroneuromyography findings.
3. The use of evoked potentials—particularly MEPs, cauda equina conduction time, segmental tibial nerve SEPs and L5 dermatomal SEPs.
4. The application of paraspinal sEMG during gait analysis and further advanced electrodiagnostic techniques in the diagnosis of LSCS.

The discussion will address these domains and compare to neurophysiological recommendations from the 2011 NASS guideline^25^ and those from the 2020 Expert Consensus.^31^

### Needle EMG in LSCS

Electrophysiological assessment of LSCS is challenging due to chronic degenerative changes and often dynamic neural compression.^25^ Anatomically, LSCS typically impinges on the spinal nerve or its roots at neuroforaminal or lateral recess levels, causing radiculopathy.^47^

Lumbosacral nerve roots, leaving the spinal cord at segment L1/2, may be compressed within the central canal before the nerve laterally exits the spine through the corresponding neuroforamen. Therefore, central stenosis can compress nerve roots at different levels, producing a pattern of multilevel and/or bilateral radiculopathy, and complicating the precise localization of the lesion.^25,48,49^ In severe cases, this may progress to cauda equina involvement.^50^

Needle EMG remains a highly specific tool for detecting radiculopathy with axonal damage.^50^ Denervation changes, as described by Wilbourn and Aminoff, are key indicators of radicular injury, especially in neuroforaminal or lateral recess compression.^48^ These signs are particularly useful when clinical symptoms are unilateral and correlate with root-level motor or sensory deficits. However, overlapping nerve root innervation can limit the sensitivity of needle EMG in early or mild cases. In cases of central canal stenosis where multiple roots are compressed, EMG abnormalities often affect several myotomes, including paraspinal and pelvic floor muscles, reflecting widespread neurogenic involvement.^49^

### The Correlation Between Clinical Symptoms, Radiologic, and Electroneuromyography Findings

For patients with a history and physical examination findings consistent with degenerative lumbar spinal stenosis, MRI is the most appropriate noninvasive test for confirming the presence of anatomic narrowing of the spinal canal or nerve root impingement.^25^

While Park *et al* ^37^ found a strong association between severe RNRs on MRI and abnormal ASAs on EMG, Rustom *et al* ^39^ reported no significant correlation between MRI severity and EMG-confirmed radiculopathy. Park *et al* ^40^ demonstrated that upper LSCS can result in radiculopathy at lower lumbar levels. These discrepancies between the anatomic site of stenosis and clinical or EMG findings highlight the importance of recognizing that LSCS can have effects that extend beyond the immediate anatomic level. Other studies also reflect variability: Matsukura *et al* ^41^ and Cai *et al* ^44^ reported a high rate of pathologic EMG findings in patients with radiographic LSCS, whereas Lin *et al* ^45^ found needle EMG-confirmed radiculopathy in only 27% of individuals with symptoms and stenosis on MRI. Taken together, these findings suggest that needle EMG can be valuable for diagnosing LSCS, particularly for confirming radiculopathy and evaluating the functional integrity of nerve roots, as its correlation with MRI findings is not linear.

### The Current Evidence for Paraspinal Mapping With Needle EMG

The current electrodiagnostic reference standard for confirming LSCS in patients presenting with mild to moderate symptoms and corresponding radiographic evidence is needle EMG with paraspinal mapping. According to the 2011 NASS guidelines^25^ and the latest 2020 expert consensus,^31^ this approach carries a Grade B recommendation. Paraspinal mapping provides valuable insight into chronic denervation and reinnervation changes in paraspinal muscles. Key studies by Haig *et al* (2005) and Yagci *et al* (2009) have substantiated its diagnostic utility.^24,51^

Haig *et al* ^26^ conducted a prospective comparative study evaluating the sensitivity and specificity of paraspinal mapping for diagnosing LSCS. EMG Paraspinal mapping of >4 segments has 100% specificity and 30% sensitivity for stenosis compared with either back pain or asymptomatic patients. A composite limb and paraspinal fibrillation score had an 87.5% specificity and a 47.8% sensitivity. Yagci *et al* ^27^ demonstrated in a prospective study that lumbar paraspinal mapping showed a high sensitivity of 96.8% and specificity of 92.3% in identifying patients with clinical and radiologic LSCS, outperforming limb needle EMG in reflecting nerve root pathology.

Despite sufficient evidence and guideline recommendations, routine clinical use of paraspinal mapping remains limited.^51^ This may reflect concerns over its invasive nature, operator dependence, potential false positives in post-surgical patients, and the absence of recent studies reaffirming its relevance. Given the procedure’s diagnostic yield and endorsement from clinical guidelines, this disconnect between evidence and implementation highlights the need for renewed investigation and education regarding its appropriate use.

### Surface EMG and Advanced Electrophysiological Methods

Over the past five years, research on paraspinal musculature in LSCS has increasingly focused on sEMG, particularly with the integration of wearable sensor technologies. This shift reflects growing interest in noninvasive diagnostic tools capable of detecting early functional impairments before structural pathology becomes apparent.^52–54^ sEMG has shown potential as a supplementary diagnostic modality for LSCS.^55^

Nüesch *et al* ^33^ observed increased activation of the multifidus, erector spinae and Gmed in LSCS patients. Similar findings were observed in patients with lumbar disc degeneration.^56^ These findings likely reflect the compensatory hyperactivation required to maintain postural stability in the presence of paraspinal muscle dysfunction.^57–59^ Kääriäinen *et al* evaluated paraspinal sEMG responses after decompression surgery and noted that while decompression surgery offers short-term proprioceptive muscle improvement, lasting paraspinal muscle damage may limit long-term monitoring utility.^60^

Surface EMG is a promising, noninvasive alternative for measuring muscle activation in the paraspinal and abductor muscles, complementing dynamic gait analysis and sensor-based kinematic evaluations.

Advanced electromyographic techniques, including MUNIX, and analysis of serum and epidural biomarkers in association with EMG signs of radiculopathy as well as functional and pain outcomes, have shown potential in the evaluation of LSCS. The integration of sEMG with NIRS has also shown good potential. While these methods provide valuable insights into neuromuscular function, their clinical applicability remains exploratory. Large-scale, controlled studies are needed to confirm their diagnostic utility and to develop standardized protocols for routine use in LSCS.^44–46^

## CONCLUSION

There has been increasing research into combining surface EMG with gait assessments. This interesting approach enables noninvasive neurophysiological evaluation while walking—*i.e.*, when symptoms commonly occur—with clinical utility yet to be determined. Paraspinal mapping has been considered the gold standard for diagnosing LSCS and was recommended as a reference in prior guidelines. However, there were no further recent studies on paraspinal mapping, potentially indicating a shift to alternative methods. There is still limited evidence to support other traditional electrophysiological techniques for diagnosing LSCS specifically, while needle EMG is established to detect radiculopathy. There is promise in comparing noninvasive against invasive EMG for improving the quantification of neural damage in LSCS.

Key Points

- Lumbar spine MRI is the standard imaging modality for detecting spinal canal narrowing but it correlates poorly with clinical symptoms.
- Electrophysiological methods may provide complementary information on neural dysfunction.
- Needle electromyography (EMG) is an established method to detect radiculopathy, while the sensitivity for detecting lumbar spinal stenosis remains limited.
- Recent electrophysiological studies focused on kinematic gait analysis combined with surface EMG, conventional needle EMG, evoked potentials, and exploratory techniques.
- There is increasing interest in gait analysis combined with surface EMG, representing a novel noninvasive functional testing avenue in lumbar stenosis.

## References

[1] Takahashi K Kagechika K Takino T Matsui T Miyazaki T Shima I . Changes in epidural pressure during walking in patients with lumbar spinal stenosis. Spine (Phila Pa 1976). 1995;20:2746–9. 8747254 10.1097/00007632-199512150-00017

[2] Lurie J Tomkins-Lane C . Management of lumbar spinal stenosis. BMJ. 2016;352:h6234. 26727925 10.1136/bmj.h6234 PMC6887476

[3] Jinkins JR . Gd-DTPA enhanced MR of the lumbar spinal canal in patients with claudication. J Comput Assist Tomogr. 1993;17:555–62. 8331225 10.1097/00004728-199307000-00007

[4] Katz JN Harris MB . Clinical practice. Lumbar spinal stenosis.. N Engl J Med. 2008;358:818–25. 18287604 10.1056/NEJMcp0708097

[5] Binder DK Schmidt MH Weinstein PR . Lumbar spinal stenosis. Semin Neurol. 2002;22:157–66. 12524561 10.1055/s-2002-36539

[6] Yabuki S Otani K Sekiguchi M . Prevalence of lumbar spinal stenosis, using the diagnostic support tool, and correlated factors in Japan: a population-based study. J Orthop Sci. 2013;18:893–900. 23963588 10.1007/s00776-013-0455-5 PMC3838585

[7] Jensen RK Jensen TS Koes B Hartvigsen J . Prevalence of lumbar spinal stenosis in general and clinical populations: a systematic review and meta-analysis. Eur Spine J. 2020;29:2143–2163. 32095908 10.1007/s00586-020-06339-1

[8] Jensen RK Harhangi BS Huygen F Koes B . Lumbar spinal stenosis. BMJ. 2021;373:n1581. 34187838 10.1136/bmj.n1581

[9] Zaina F Tomkins-Lane C Carragee E Negrini S . Surgical versus nonsurgical treatment for lumbar spinal stenosis. Spine (Phila Pa 1976). 2016;41:E857–E868. 27128388 10.1097/BRS.0000000000001635

[10] Ravindra VM Senglaub SS Rattani A . Degenerative lumbar spine disease: estimating global incidence and worldwide volume. Global Spine J. 2018;8:784–794. 30560029 10.1177/2192568218770769 PMC6293435

[11] Katz JN Dalgas M Stucki G . Degenerative lumbar spinal stenosis. Diagnostic value of the history and physical examination. Arthritis Rheum. 1995;38:1236–41. 7575718 10.1002/art.1780380910

[12] Jensen RK Lauridsen H H Andresen A D K Mieritz R M Schiottz-Christensen B Vach W . Diagnostic screening for lumbar spinal stenosis. Clin Epidemiol. 2020;12:891–905. 32904080 10.2147/CLEP.S263646 PMC7450213

[13] Saifuddin A . The imaging of lumbar spinal stenosis. Clin Radiol. 2000;55:581–94. 10964728 10.1053/crad.2000.0223

[14] Wildermuth S Zanetti M Duewell S . Lumbar spine: quantitative and qualitative assessment of positional (upright flexion and extension) MR imaging and myelography. Radiology. 1998;207:391–8. 9577486 10.1148/radiology.207.2.9577486

[15] Lim YS Mun JU Seo MS . Dural sac area is a more sensitive parameter for evaluating lumbar spinal stenosis than spinal canal area: a retrospective study. Medicine. 2017;96:e9087. 29245329 10.1097/MD.0000000000009087 PMC5728944

[16] Andreisek G Hodler J Steurer J . Uncertainties in the diagnosis of lumbar spinal stenosis. Radiology. 2011;261:681–4. 22095990 10.1148/radiol.11111086

[17] Boden SD Davis DO Dina TS Patronas NJ Wiesel SW . Abnormal magnetic-resonance scans of the lumbar spine in asymptomatic subjects. A prospective investigation. J Bone Joint Surg Am. 1990;72:403–8. 2312537

[18] Denard PJ Holton KF Miller J . Lumbar spondylolisthesis among elderly men: prevalence, correlates, and progression. Spine (Phila Pa 1976). 2010;35:1072–8. 20393398 10.1097/BRS.0b013e3181bd9e19 PMC2903965

[19] Suri P Rainville J Kalichman L Katz JN . Does this older adult with lower extremity pain have the clinical syndrome of lumbar spinal stenosis? JAMA. 2010;304:2628–36. 21156951 10.1001/jama.2010.1833 PMC3260477

[20] Tong HC Carson JT Haig AJ . Magnetic resonance imaging of the lumbar spine in asymptomatic older adults. J Back Musculoskelet Rehabil. 2006;19(2-3):67–72.

[21] Kalra K Chahal R Kumar M . Lumbar canal stenosis: a prospective clinicoradiologic analysis. J Neurol Surg: Central Eur Neurosurg. 2020;81:387–391. 10.1055/s-0039-1698393 32107754

[22] Kajdi GW Marth T Choi JA Farshad M Sutter R . Diagnostic value of a coronal STIR sequence in conjoined lumbar nerve root detection: an MRI accuracy study. Skeletal Radiol. 2025;54:2157–2168. 40369228 10.1007/s00256-025-04945-y PMC12361294

[23] Adamova B Vohanka S Dusek L . Differential diagnostics in patients with mild lumbar spinal stenosis: the contributions and limits of various tests. Eur Spine J. 2003;12:190–6. 12709857 10.1007/s00586-002-0503-x PMC3784841

[24] Glocker FLR . S2k-Leitlinie, 2018; in: Deutsche Gesellschaft für Neurologie (Hrsg.), Leitlinien für Diagnostik und Therapie in der Neurologie. Accessed March 7, 2025. www.dgn.org/leitlinien

[25] Kreiner S SW Baisden J, . Evidence-Based Clinical Guidelines for Multidisciplinary Spine Care. North American Spine Society; 2011. Accessed March 7, 2025. https://www.spine.org/Portals/0/Assets/Downloads/ResearchClinicalCare/Guidelines/LumbarStenosis.pdf

[26] Haig AJ Tong HC Yamakawa KSJ . The sensitivity and specificity of electrodiagnostic testing for the clinical syndrome of lumbar spinal stenosis. Spine (Phila Pa 1976). 2005;30:2667–76. 16319753 10.1097/01.brs.0000188400.11490.5f

[27] Yagci I Gunduz OH Ekinci G Diracoglu D Us O Akyuz G . The utility of lumbar paraspinal mapping in the diagnosis of lumbar spinal stenosis. Am J Phys Med Rehabil. 2009;88:843–51. 19661776 10.1097/PHM.0b013e3181b333a9

[28] Dillingham TR Annaswamy TM Plastaras CT . Evaluation of persons with suspected lumbosacral and cervical radiculopathy: electrodiagnostic assessment and implications for treatment and outcomes (Part I). Muscle Nerve. 2020;62:462–473. 32557709 10.1002/mus.26997

[29] Dillingham TR Annaswamy TM Plastaras CT . Evaluation of persons with suspected lumbosacral and cervical radiculopathy: electrodiagnostic assessment and implications for treatment and outcomes (Part II). Muscle Nerve. 2020;62:474–484. 32564381 10.1002/mus.27008

[30] Page M J Moher D Bossuyt P M . PRISMA 2020 explanation and elaboration: updated guidance and exemplars for reporting systematic reviews. BMJ. 2021;372:n160. doi:10.1136/bmj.n160 33781993 PMC8005925

[31] Zileli M Crostelli M Grimaldi M . Natural course and diagnosis of lumbar spinal stenosis: WFNS Spine Committee Recommendations. World Neurosurg X. 2020;7:100073. 32613187 10.1016/j.wnsx.2020.100073 PMC7322797

[32] The Risk Of Bias In Non-randomized Studies – of Interventions, Version 2 (ROBINS-I V2). Accessed March 7, 2025. https://www.riskofbias.info/welcome/robins-i-v2

[33] Nüesch C Mandelli F Przybilla P Schären S Mündermann A Netzer C . Kinematics and paraspinal muscle activation patterns during walking differ between patients with lumbar spinal stenosis and controls. Gait Posture. 2023;99:44–50. 36327537 10.1016/j.gaitpost.2022.10.017

[34] Urbanschitz L Nüesch C Schären S Mandelli F Mündermann A Netzer C . Walking stress-induced changes in gait patterns and muscle activity: Patients with lumbar spinal stenosis versus asymptomatic controls. Gait Posture. 2024;114:55–61. 39243529 10.1016/j.gaitpost.2024.08.083

[35] Kim JJ Cho H Park Y Jang J Kim J W Ryu JS . Biomechanical influences of gait patterns on knee joint: Kinematic & EMG analysis. PLoS One. 2020;15:e0233593. 32470052 10.1371/journal.pone.0233593 PMC7259630

[36] Shin SS Yoo WG . The effects of differences on sagittal kinematics and muscle coordination during step up and step down in patients with spinal stenosis. J Back Musculoskelet Rehabil. 2021;34:453–459. 33492274 10.3233/BMR-200118

[37] Park S Hong SH Chung SG Kim K . Redundant nerve roots on magnetic resonance imaging can predict ongoing denervation in patients with lumbar spinal stenosis. Muscle Nerve. 2024;69:691–698. 38545741 10.1002/mus.28094

[38] Marques CJ Hillebrand H Papavero L . The clinical significance of redundant nerve roots of the cauda equina in lumbar spinal stenosis patients: a systematic literature review and meta-analysis. Clin Neurol Neurosurg. 2018;174:40–47. 30205275 10.1016/j.clineuro.2018.09.001

[39] Rustom DH Yan A Seidel GK . Electrodiagnostic confirmation of lumbar radiculopathy and its association with lumbar central canal stenosis and neuroforaminal stenosis. Cureus. 2024;16:e69993. 39445271 10.7759/cureus.69993 PMC11497861

[40] Park JH Chung SG Kim K . Electrodiagnostic characteristics of upper lumbar stenosis: discrepancy between neurological and structural levels. Muscle Nerve. 2020;61:580–586. 32096875 10.1002/mus.26844

[41] Matsukura K Hokkoku K Mukai T . Tibial nerve SEPs in diagnosing lumbar spinal stenosis: the utility of segmental evaluation using P15 and N21. Clin Neurophysiol Pract. 2023;8:49–57. 37008279 10.1016/j.cnp.2023.03.001 PMC10064344

[42] Yang DC Lee HJ Park JW . Association between latency of dermatomal sensory-evoked potentials and quantitative radiologic findings of narrowing in lumbar spinal stenosis. Ann Rehabil Med-Arm. 2020;44:353–361. 10.5535/arm.19164 PMC7655228 32986946

[43] Nagao Y Imajo Y Funaba M . Relationship between cauda equina conduction time and type of neurogenic intermittent claudication due to lumbar spinal stenosis. J Clin Neurophysiol. 2020;37:62–67. 31335564 10.1097/WNP.0000000000000607

[44] Cai H Kroll M Annaswamy T . Motor unit number index in evaluating patients with lumbar spinal stenosis. Am J Phys Med Rehabil. 2021;100:966–971. 33433110 10.1097/PHM.0000000000001681

[45] Lin CK Borresen A Kroll M Annaswamy TM . Predicting response to epidural steroid injections for lumbar spinal stenosis with biomarkers and electromyography. PM R. 2020;12:663–670. 31659847 10.1002/pmrj.12272

[46] Ekström L Zhang Q Abrahamson J . A model for evaluation of the electric activity and oxygenation in the erector spinae muscle during isometric loading adapted for spine patients. J Orthop Surg Res. 2020;15:155. 32303232 10.1186/s13018-020-01652-3 PMC7165389

[47] Deyo RA Weinstein JN . Primary care - Llow back pain. N Engl J Med. 2001;344:363–370. 11172169 10.1056/NEJM200102013440508

[48] Wilbourn AJ Aminoff MJ . AAEM minimonograph 32: the electrodiagnostic examination in patients with radiculopathies. Am Assoc Electrodiagnostic Med Muscle Nerve. 1998;21:1612–31. 10.1002/(sici)1097-4598(199812)21:12<1612::aid-mus2>3.0.co;2-0 9843062

[49] Kimura J . Electrodiagnosis in diseases of nerve and muscle: Principles and practice, Fourth edition. New York: Oxford University Press; 2013:644–649.

[50] Taruilli AW Raynor EM . Lumbosacral radiculopathy. Neurol Clin. 2007;25:387. 17445735 10.1016/j.ncl.2007.01.008

[51] Kreuzschmerz Spezifischer . S2k-Leitlinie der Deutschen Gesellschaft für Orthopädie und Unfallchirurgie e.V. https://register.awmf.org/assets/guidelines/187-059l_S2k_Spezifischer-Kreuzschmerz_2024-08.pdf (Consulted on 03.07.2025).

[52] Suo M Zhou L Wang J . The application of surface electromyography technology in evaluating paraspinal muscle function. Diagnostics. 2024;14:1086. 38893614 10.3390/diagnostics14111086 PMC11172025

[53] Olson MW . Trunk muscle activation during sub-maximal extension efforts. Manual Ther. 2010;15:105–110. 10.1016/j.math.2009.08.005 19716741

[54] Kankaanpää M Laaksonen D Taimela S Kokko SM Airaksinen O Hänninen O . Age, sex, and body mass index as determinants of back and hip extensor fatigue in the isometric Sorensen back endurance test. Archiv Phys Med Rehabil. 1998;79:1069–1075. 10.1016/s0003-9993(98)90173-3 9749686

[55] Arokoski JPA Kankaanpää M Valta T . Back and hip extensor muscle function during therapeutic exercises. Archiv Phys Med Rehabil. 1999;80:842–850. 10.1016/s0003-9993(99)90237-x 10414772

[56] Miscusi M Serrao M Conte C . Spatial and temporal characteristics of the spine muscles activation during walking in patients with lumbar instability due to degenerative lumbar disk disease: evaluation in pre-surgical setting. Human Movement Sci. 2019;66:371–382. 10.1016/j.humov.2019.05.013 31153034

[57] Kuwahara W Deie M Fujita N . Characteristics of thoracic and lumbar movements during gait in lumbar spinal stenosis patients before and after decompression surgery. Clin Biomechan. 2016;40:45–51. 10.1016/j.clinbiomech.2016.10.016 27821273

[58] Goto T Sakai T Enishi T . Changes of posture and muscle activities in the trunk and legs during walking in patients with lumbar spinal stenosis after decompression surgery. A preliminary report. Gait Posture. 2017;51:149–152. 27764750 10.1016/j.gaitpost.2016.10.006

[59] Igawa T Katsuhira J Hosaka A Uchikoshi K Ishihara S Matsudaira K . Kinetic and kinematic variables affecting trunk flexion during level walking in patients with lumbar spinal stenosis. Plos One. 2018;13:e0197228. 29746537 10.1371/journal.pone.0197228 PMC5944950

[60] Kääriäinen T Taimela S Aalto T . The effect of decompressive surgery on lumbar paraspinal and biceps brachii muscle function and movement perception in lumbar spinal stenosis: a 2-year follow-up. Eur Spine J. 2016;25:789–794. 26014807 10.1007/s00586-015-4036-5

